# Overexpression of semaphorin 3A promotes tumor progression and predicts poor prognosis in hepatocellular carcinoma after curative resection

**DOI:** 10.18632/oncotarget.10104

**Published:** 2016-06-16

**Authors:** Zhi-Qiang Hu, Shao-Lai Zhou, Zheng-Jun Zhou, Chu-Bin Luo, Er-Bao Chen, Hao Zhan, Peng-Cheng Wang, Zhi Dai, Jian Zhou, Jia Fan, Xiao-Wu Huang

**Affiliations:** ^1^ Liver Cancer Institute, Zhongshan Hospital, Fudan University, Key Laboratory of Carcinogenesis and Cancer Invasion, Fudan University, Ministry of Education, Shanghai 200032, China

**Keywords:** hepatocellular carcinoma, semaphorin 3A, tumor-associated macrophages, prognosis

## Abstract

The semaphorins were originally identified as having roles as guidance cues during neural development. Class 3 semaphorins are involved in cancer progression. However, the roles of class 3 semaphorins in hepatocellular carcinoma (HCC) are unknown. We examined the expression levels of class 3 semaphorins in HCC cell lines with different metastatic potential and in carcinoma tissue samples. The results indicated that Semaphorin 3A expression was up-regulated in metastatic cell lines and in samples from patients with tumor recurrence. Cell functional studies revealed that Semaphorin 3A promoted HCC cell proliferation, migration, and invasion. Animal studies indicated that Semaphorin 3A overexpression enhanced tumor growth and lung metastasis. Semaphorin 3A also acted as a chemoattractant involved in direct recruitment of macrophages in vitro, and facilitated tumor-associated macrophage (TAM) infiltration *in vivo*. Multivariate analysis revealed that Semaphorin 3A expression alone, or combined with the number of TAMs, can be an independent predictor for overall survival time and time to recurrence. Overall, the results suggested that Semaphorin 3A increased TAM infiltration and promoted HCC progression. Semaphorin 3A expression alone, or combined with the number of TAMs, is a new prognostic factor and potential target for the treatment of HCC.

## INTRODUCTION

Survival rates of patients with hepatocellular carcinoma (HCC) have increased as the result of advances in surgery and the use of transarterial chemoembolization (TACE) or other targeted therapies. However, the ratios of recurrence and metastasis after curative resection have not decreased [1, 2]. Therefore, it is essential to understand the mechanisms that increase metastasis and recurrence of this third most common cause of cancer-related death [3].

Semaphorins and the semaphorin receptors (i.e., plexins and neuropilins) were initially found in the embryonic nervous system. They usually participate in neuronal network and neurite outgrowth establishment; they also may act as chemorepellents or chemoattractants [4, 5]. Semaphorins are not only involved in the developing nervous system, but are also expressed in a wide range of non-neural cells and modulate numerous physiological and pathological processes [6–9]. Some members of class 3 semaphorins (e.g., Sema3C, Sema3E) are up-regulated in some types of cancers [10, 11]. However, some class 3 semaphorins members also have tumor suppressor activity. For example, Sema3B and Sema3F are often down-regulated in liver and ovarian cancer respectively [12, 13]. This classification is ambiguous, however, because some semaphorins have anti-tumoral and pro-tumoral characteristics. Sema3A is down-regulated in breast cancer [14], meningioma [15] and mesothelioma [16] patients; it is overexpressed in pancreatic cancer patients with poor outcomes [17].

To identify the functions of class 3 semaphorins in HCC, we examined the expression of this group in cell lines with different metastatic potential and in tumor tissue samples. We found that one class 3 semaphorins family member, Sema3A, had a crucial role in carcinoma development. Our results indicated that Sema3A promoted HCC cell proliferation, migration, and invasion. We also found that TAM infiltration was altered by changes in the expression of Sema3A. Sema3A expression was strongly associated with tumor recurrence and predicted a poorer post-surgical prognosis for patients.

## RESULTS

### Sema3A expression was associated with the metastatic potential of HCC cells and patient recurrence

**Figure 1:**
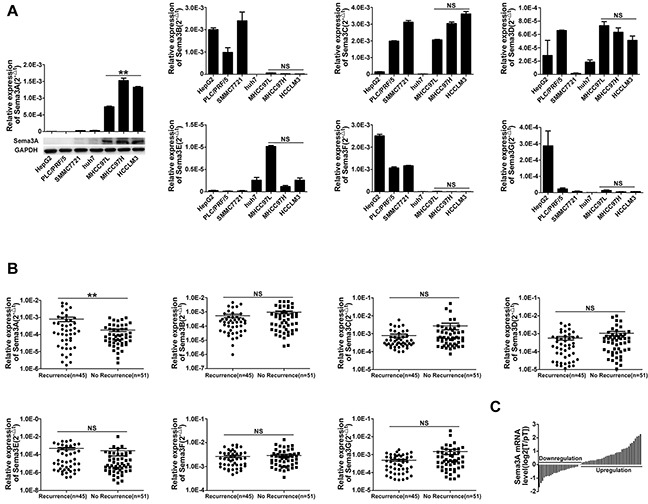
Class 3 semaphorin (Sema3A, 3B, 3C, 3D, 3E, 3F, 3G) expression in seven cell lines (HepG2, PLC/PRF/5, SMMC7721, Huh7, MHCC97L, MHCC97H, and HCCLM3) with different invasive potential (low and high), and in HCC tissue **A.** qRT-PCR and western blot confirmed different class 3 Semaphorin mRNA expression in the cell lines. The mean (± standard deviation (SD)) values from three independent experiments are indicated, **p<0.01, compared with low invasive potential cell lines (HepG2, PLC/PRF/5, SMMC7721 and Huh7). **B.** Results for qRT-PCR analysis of class 3 Semaphorin expression in 96 tumors taken from patients with, or without, tumor recurrence, **p<0.01. **C.** qRT-PCR analysis of Sema3A expression in 63 paired samples taken from HCCs and from surrounding tissue.

To examine the roles of class 3 semaphorins in HCC progression, we first measured the mRNA level of class 3 semaphorins (Sema3A, 3B, 3C, 3D, 3E, 3F, and 3G) in seven human cell lines with varying potential for metastasis (Figure 1A). The results indicated that as the invasion and metastasis of HCC cell lines increased, only Sema3A expression was significantly increased. Compared with the low metastatic cell lines (HepG2, PLC/PRF/5, Huh7, and SMMC7721), both the mRNA and protein levels of Sema3A in the highly metastatic cell lines (MHCC97L, MHCC97H and HCCLM3) were much higher (P<0.01). We also found that unlike other Sema3s, there were higher Sema3A mRNA levels in patients with recurrence, compared with patients without recurrence (p<0.01; Figure 1B). In addition, the mRNA level of Sema3A in nearly 60% of the HCC tumors was significantly evaluated compared with the matched peritumoral tissues (p<0.05; Figure 1C). These results suggested that Sema3A expression is frequently up-regulated in human HCCs, particularly in HCC patients with recurrence. This upregulation might have a significant role in HCC progression.

### Sema3A enhanced the ability of HCC cells to proliferate, migrate, and invade

**Figure 2:**
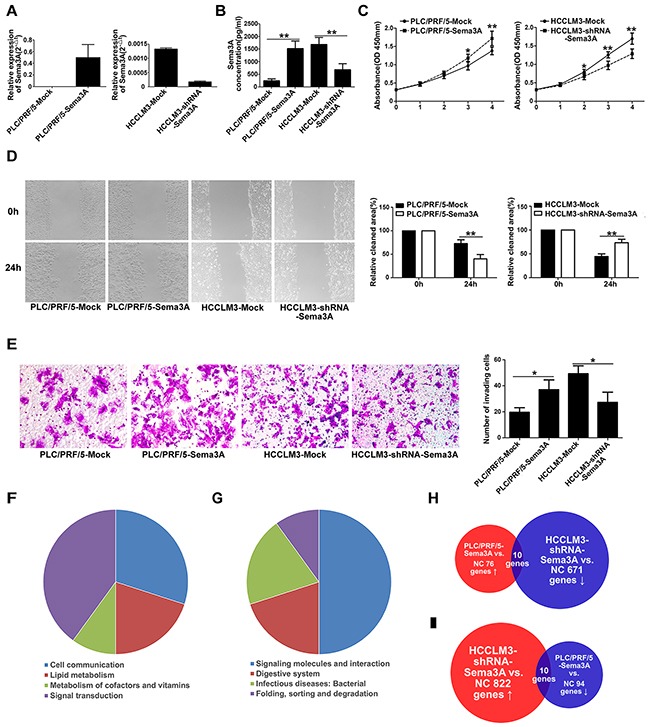
Sema3A promoted proliferation, migration, and invasion of HCC cells in vitro and promoted gene changes between stably transfected and parent cells **A.** Sema3A expression was confirmed using qRT-PCR analysis of stably transfected and of parent cells. **B.** ELISA results indicated the presence of significantly greater levels of Sema3A proteins in PLC/PRF/5-Sema3A and HCCLM3-Mock cells, compared with PLC/PRF/5-Mock and HCCLM3-shRNA-Sema3A. The mean (± SD) values from three independent experiments are presented, **p<0.01. **C.** CCK8 assay was used to detect cell proliferation, *p<0.05; **p<0.01. **D.** The results for wound-healing migration assays and the relative quantification of percent open area are presented. Results shown are mean (±SD) values from three independent experiments, **p<0.01. Magnification, ×200. **E.** Transwell Matrigel invasion assays were used to test invasive behavior. The results are mean (±SD) values from three independent experiments, *p<0.05. Magnification, ×200. **F.** The results are from four groups that included 10 genes that were present in the up-regulated (ratio, >2.0) genes in the PLC/PRF/5-Sema3A cells and in the down-regulated (ratio, <0.5) genes in HCCLM3-shRNA-Sema3A cells. **G.** There were also four groups that included 10 genes that were down-regulated by Sema3A overexpression and also up-regulated by Sema3A knockdown. **H-I.** PLC/PRF/5-Sema3A cells compared with PLC/PRF/5-Mock cells: genes that were up-regulated (76; ratio, 2.0) and down-regulated (94; ratio, 0.5). HCCLM3-shRNA-Sema3A cells compared with HCCLM3-Mock cells: genes that were up-regulated (822; ratio, 2.0) and genes that were down-regulated (671; ratio, 0.5).

To verify that Sema3A expression was stably up- and down-regulated after cellular transfection, we used qRT-PCR (Figure 2A). Proliferation assay results indicated that, when Sema3A expression was knocked down by shRNA in HCCLM3 cells, proliferation was suppressed at 48 hours (p<0.05). Compared with PLC/PRF/5-Mock cells, the proliferation ability of PLC/PRF/5-Sema3A cells was enhanced (p<0.05; Figure 2C). Microscopic examination of the wound-healing migration assays revealed that HCCLM3-shRNA-Sema3A cells showed a significant suppression of cell migration, compared with HCCLM3-Mock cells (p<0.01; Figure 2D). Similarly, compared with PLC/PRF/5-Mock cells, wound closure rate was greater in PLC/PRF/5-Sema3A cells (p<0.01; Figure 2D). The results of the invasive assays indicated that there were more invasive HCCLM3-Mock cells than HCCLM3-shRNA-Sema3A cells (p<0.05). There were also significantly more invasive PLC/PRF/5-Sema3A cells than there were PLC/PRF/5-Mock cells (p<0.05; Figure 2E).

### Gene expression profile based on microarray analysis

To understand the role of Sema3A in HCC cell progression, we examined genes perturbed by Sema3A overexpression and knockdown. The microarray analysis results indicated that 170 genes were differentially expressed between the PLC/PRF/5-Sema3A and the PLC/PRF/5-mock cells. Of these genes, 76 were up-regulated (ratio>2.0) and 94 were down-regulated (ratio<0.5). A total of 1493 genes were differentially expressed between the HCCLM3-shRNA-Sema3A and the HCCLM3-mock cells. Of these genes, 822 were up-regulated (ratio, 2.0) and 671 down-regulated (ratio, 0.5). Ten genes (CLDN16, CLDN2, MRAS, AGPAT3, CEL, PHOSPHO2, ITGA3, PDGFD, PPP2R5C, SGK2) could be found among both the up-regulated genes in the PLC/PRF/5-Sema3A cells and the down-regulated genes in the HCCLM3-shRNA-Sema3A cells (Figure 2H). These genes were involved in tumor metastasis and regulation of cell migration. According to the Kyoto Encyclopedia of Genes and Genomes, these genes can be categorized into four groups based on the significant signaling pathways (tight junction, glycerolipid metabolism, vitamin B6 metabolism, and PI3K-Akt signaling pathways) (Figure 2F). These pathways are involved in carcinogenesis and metastasis [18–24]. Ten genes were down-regulated by Sema3A overexpression and also up-regulated by Sema3A knockdown (Figure 2I). Kyoto Encyclopedia of Genes and Genomes analysis revealed that these genes are involved in molecular signaling and interaction, the digestive system, infectious disease, and the sulfur relay system (Figure 2G).

### Sema3A was a chemoattractant for macrophages in vitro

**Figure 3:**
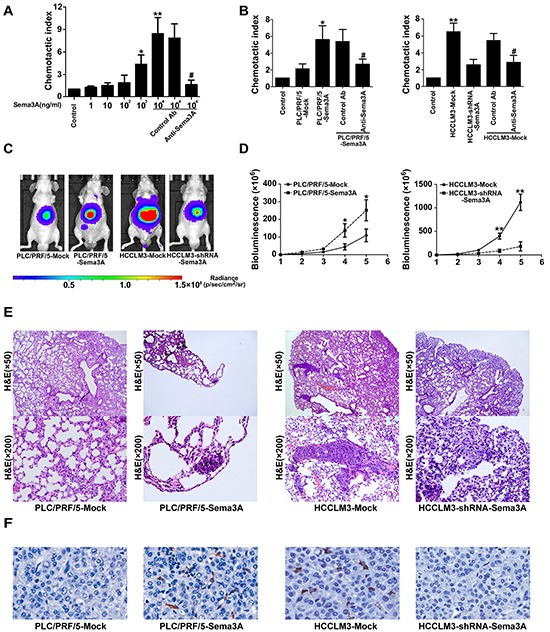
Sema3A promoted tumor growth and metastasis by recruiting macrophages (nude mouse model) **A.** Recombinant human Sema3A recruited macrophages from HCC patients. This response was abrogated by anti-Sema3A antibody. *p<0.05; **p<0.01 compared with control; #P < 0.05 when compared with control antibody (Ab). **B.** Transwell assay and chemotactic migration of macrophages was determined after exposure to conditioned medium derived from different HCC cells. When we treated the CM of HCC cells with a high level of Sema3A with anti-Sema3A antibody, chemotactic response of macrophage decreased. Results shown are mean (±SD) values from three independent experiments, *p<0.05; **p<0.01 compared with control; #P < 0.05 when compared with control Ab. **C.** Representative bioluminescence images of orthotopic tumor at day 35. The color scale bar depicts the photon flux emitted from these tumors, n=8. **D.** Growth curves of tumors in xenograft nude mouse models, assessed by bioluminescent signals, n=8, *p<0.05; **p<0.01. **E.** Representative views of H&E stain results of metastatic nodules (lung tissue) from each group, n=8. **F.** Representative images of tumor samples from xenograft nude mouse models stained with F4/80. Magnification, ×200.

It has been showed that Sema3A can recruit macrophages to hypoxic niches [25]. We confirmed that recombinant human Sema3A can recruit macrophages. When treated with anti-Sema3A, this response was abrogated (Figure 3A). Next we examined whether HCC cells can recruit human macrophages in vitro through Sema3A. We collected CM from PLC/PRF/5-mock cells, PLC/PRF/5-Sema3A cells, HCCLM3-mock cells, and HCCLM3-shRNA-Sema3A cells, and detected Sema3A protein levels using enzyme-linked immuno-sorbent assay (Figure 2B). We found that compared with PLC/PRF/5-mock cells, Sema3A secretion was increased by PLC/PRF/5-Sema3A. Secretion was significantly decreased in HCCLM3-shRNA-Sema3A cells, compared with HCCLM3-Mock cells. Using a transwell system to examine the effect of Sema3A on macrophage chemotaxis, we found that macrophage migration was enhanced by the CM from PLC/PRF/5-Sema3A cells and was suppressed by the CM from HCCLM3-shRNA-Sema3A cells, compared with the CM from control cells (Figure 3B). In addition, when we treated the CM derived from HCC cells with a high level of Sema3A (PLC/PRF/5-Sema3A and HCCLM3-Mock) with anti-Sema3A neutralizing antibody, the chemotactic response of macrophage decreased. Thus, CM of HCC cells with a high level of Sema3A (PLC/PRF/5-Sema3A and HCCLM3-Mock) contributed to a significant increase in macrophage chemotaxis. However, CM from HCC cells with low Sema3A expression (PLC/PRF/5-mock and HCCLM3-shRNA-Sema3A) only induced a small increase.

### Sema3A increased TAM infiltration and promoted HCC progression in vivo

We also investigated the effect of Sema3A using an orthotopic nude mouse liver cancer model. All cell lines (5×10^6^) were suspended in 100 μL serum-free DMEM and Matrigel (1:1). Each mouse received a subcutaneous injection of cells into the upper left flank area. The developing subcutaneous tumor was removed when it was approximately 1 cm in length (approximately 4 weeks post-injection) and was cut into small pieces (2×2×2 mm^3^). One piece was then transplanted into the liver of each mouse. The results indicated that the tumor volumes derived from the HCCLM3-shRNA-Sema3A xenografts models were significantly smaller than that of the xenografts models derived from HCCLM3-Mock cells. Similarly, the tumor volumes of the PLC/PRF/5-Sema3A-derived xenografts models were visibly larger than that of the PLC/PRF/5-Mock-derived tumors (Figure 3C). The results from the bioluminescence imaging studies were similar (Figure 3D).

Increased rates of pulmonary metastases (100%) were observed in the HCCLM3-Mock, compared with the HCCLM3-shRNA-Sema3A mice (37.5%). The greater the Sema3A expression, the greater the number of nodules of each grade that were metastatic (Figure 3E). The rate of pulmonary metastases in the PLC/PRF/5-Sema3A mice was 75%, which was significantly higher compared with the rate in the PLC/PRF/5-Mock mice (0%, Figure 3E). In the HCCLM3-Mock-derived and PLC/PRF/5-Sema3A-derived xenografts, the infiltration of F4/80+ TAMs was, greater than the infiltration that occurred in the HCCLM3-shRNA-Sema3A-derived and the PLC/PRF/5-Mock-derived xenografts, respectively (Figure 3F).

### Sema3A expression and combined expression of Sema3A and TAMs correlated with patient outcome

**Figure 4:**
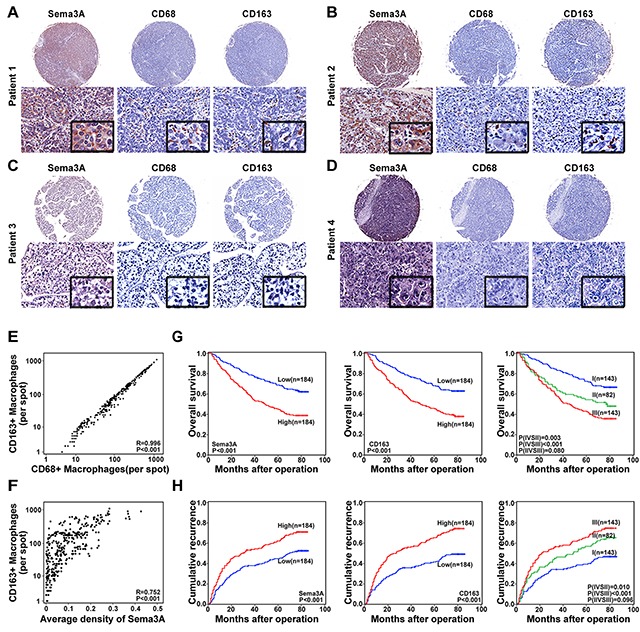
Expression and prognostic value of Sema3A and TAMs in carcinoma samples (cohort 3, n=368) **A–D.** Representative HCC tumor samples, expression of Sema3A and TAM (CD68 and CD163). (A–B) High levels of expression, Sema3A and TAM. (C–D) Low levels of expression, Sema3A and TAM. **E.** Scatterplot results, statistically significant positive correlation, intratumoral CD68 and intratumoral CD163 levels in tumor tissue. **F.** Scatterplot results, statistically significant positive correlation, Sema3A and intratumoral CD163 levels in tumor tissue. **G–H.** Kaplan-Meier analysis results, Sema3A and intratumoral CD163 prognostic values. I, Sema3A ^low^/intratumoral CD163^low^; II, Sema3A^low^/intratumoral CD163^high^ and Sema3A^high^/intratumoral CD163^low^; III, Sema3A^high^/intratumoral CD163^high^.

Based on the in vitro and in vivo findings, we assessed the potential relationships between Sema3A and clinical characteristics. Using immunohistochemical staining, we detected the expression of Sema3A and intratumoral macrophages in a tissue microarray that was constructed using primary tumors from 368 HCC patients. The results are presented in Figure 4A-4D. We observed a significant correlation between the expression of Sema3A and the number of intratumoral macrophages, especially M2 tumor-associated macrophages (CD163+ macrophages) (Figure 4E-4F). Sema3A level was positively correlated with tumor size (p=0.005), tumor number (p=0.044), tumor encapsulation (p=0.046) and tumor-node-metastasis staging (p=0.018) (Table 1). A total of 48.6% (179/368) of the patients had died and 60.3% (222/368) suffered a recurrence by the last follow-up date of 15 March 2013. The 1-, 3-, and 5-year OS rates in Cohort 3 were 88.3%, 68.7%, and 57.0%, respectively. The cumulative recurrence rates were 27.4%, 45.7%, and 53.5%, respectively. The 1-, 3-, and 5-year survival rates of the Sema3A^low^ patients were greater compared with those of the Sema3A^high^ group (91.8% versus 84.8%, 78.8% versus 58.6%, and 68.8% versus 45.3%, respectively; Figure 4G). Similarly, compared with the Sema3A^high^ patients, the Sema3A^low^ HCC patients had a better prognosis and lower 1-, 3-, and 5-year recurrence rates (cumulative rates of 19.6% versus 35.3%, 38.0% versus 53.3%, and 44.9% versus 62.3%, respectively; Figure 4H). The correlation between TAM presence and OS time was statistically significant (p<0.001, hazard ratio (HR) =2.122) and TTR (p<0.001, HR=1.923; Table 2). Patients with high levels of Sema3A had greater TAM infiltration compared with patients with low Sema3A levels (n=368, r=0.752, p< 0.001) (Figure 4F). When we combined the Sema3A expression and TAM presence to evaluate the effect on HCC prognosis, we found that the OS rates of the Sema3A^high^/intratumoral macrophages^high^ patients at 1, 3, and 5 years were 84.6%, 57.3%, and 43.1%, respectively. However, the 1-, 3-, and 5-year OS rates of the patients with Sema3A^low^/intratumoral macrophages^low^ were 95.1%, 83.2%, and 72.5%, respectively (Figure 4G). The recurrence rates for the patients with Sema3A^high^/intratumoral macrophages^high^ were much higher, with a cumulative rate of 37.1% at 1 year, 56.7% at 3 years, and 65.9% at 5 years compared with the patients with Sema3A^low^/intratumoral macrophages^low^ (17.5%, 34.3%, and 39.4%, at 1, 3, and 5 years respectively; Figure 4H). The results of the analyses (univariate and multivariate) indicated that along with gamma glutamyl transferase and tumor differentiation, Sema3A expression and the Sema3A/ TAM co-index were independent predictors of prognosis for OS (p<0.001, HR =1.912 and p<0.001, HR =2.296) and TTR (p=0.001, HR =1.577 and p<0.001, HR =1.964; Table 2).

**Table 1:** Correlation between the factors and clinicopathologic characteristics in HCC (cohort 3, n=368)

Clinicopathological indexes		Sema3A			CD68			CD163		
		Low	High	P	Low	High	P	Low	High	P
Age(year)	≤50	70	73	0.748	74	69	0.593	74	69	0.593
	>50	114	111		110	115		110	115	
Sex	Female	31	34	0.682	33	32	0.891	33	32	0.891
	Male	153	150		151	152		151	152	
HBsAg	Negative	28	25	0.656	24	29	0.458	21	32	0.102
	Positive	156	159		160	155		163	152	
HCV	Negative	182	183	0.624*	183	182	0.624*	183	182	0.624*
	Positive	2	1		1	2		1	2	
AFP (ng/ml)	≤20	75	64	0.237	73	66	0.452	73	66	0.452
	>20	109	120		111	118		111	118	
GGT (U/L)	≤54	95	96	0.917	101	90	0.251	98	93	0.602
	>54	89	88		83	94		86	91	
Liver cirrhosis	No	27	33	0.397	33	27	0.397	31	29	0.778
	Yes	157	151		151	157		153	155	
Tumor size(cm)	≤5	125	99	0.005	129	95	0.000	127	97	0.001
	>5	59	85		55	89		57	87	
Tumor number	Single	170	158	0.044	168	160	0.180	169	159	0.094
	Multiple	14	26		16	24		15	25	
Microvascular invasion	Absence	135	118	0.111	135	118	0.111	137	116	0.018
	Present	49	65		49	65		47	68	
Tumor encapsulation	Complete	111	92	0.046	107	96	0.249	106	97	0.345
	None	73	92		77	88		78	87	
Tumor differentiation	I+II	138	138	1.0	144	132	0.149	146	130	0.054
	III+IV	46	46		40	52		38	54	
TNM stage	I	125	103	0.018	122	106	0.086	124	104	0.032
	II+III	59	81		62	78		60	80	

AFP, alpha-fetoprotein; GGT, gamma glutamyl transferase; TNM, tumor-node-metastasis.

* Fisher's exact tests; chi-square tests for all other analyses.

**Table 2:** Univariate and multivariate analyses of prognostic factors in HCC (cohort 3, n =368)

Variable	TTR		OS	
	HR (95% CI)	P	HR (95% CI)	P
Univariate analysis				
Age, year (≤50 versus >50)	1.239(0.939-1.634)	0.130	1.036(0.765-1.402)	0.820
Sex (female versus male)	1.422(0.977-2.069)	0.066	1.115(0.749-1.659)	0.593
HBsAg (negative versus positive)	1.378(0.915-2.076)	0.124	1.160(0.749-1.798)	0.506
AFP, ng/ml (≤20 versus >20)	1.340(1.016-1.767)	0.038	1.654(1.203-2.272)	0.002
GGT, U/L (≤54 versus >54)	1.702(1.305-2.220)	0.000	1.832(1.360-2.468)	0.000
Liver cirrhosis (no versus yes)	1.643(1.098-2.459)	0.016	1.459(0.934-2.280)	0.097
Tumor size, cm (≤5 versus >5)	1.515(1.162-1.976)	0.002	1.925(1.435-2.582)	0.000
Tumor number (single versus multiple)	1.652(1.128-2.418)	0.010	1.790(1.195-2.681)	0.005
Microvascular invasion (no versus yes)	1.767(1.346-2.319)	0.000	1.821(1.349-2.457)	0.000
Tumor encapsulation (complete versus none)	1.289(0.991-1.678)	0.059	1.266(0.944-1.697)	0.115
Tumor differentiation (I + II versus III + IV)	1.451(1.082-1.946)	0.013	1.757(1.282-2.406)	0.000
TNM stage (I versus II III)	1.738(1.333-2.267)	0.000	1.986(1.481-2.663)	0.000
Sema3A (low versus high)	1.663(1.274-2.172)	0.000	2.072(1.531-2.805)	0.000
Intratumor CD68 (low versus high)	1.956(1.493-2.563)	0.000	2.223(1.639-3.015)	0.000
Intratumor CD163 (low versus high)	1.923(1.469-2.519)	0.000	2.122(1.567-2.874)	0.000
Combination of Sema3A and CD163*		0.000		0.000
I versus II	1.602(1.113-2.306)	0.011	1.885(1.239-2.867)	0.003
II versus III	1.323(0.948-1.847)	0.099	1.387(0.959-2.006)	0.082
I versus III	2.125(1.558-2.899)	0.000	2.646(1.858-3.767)	0.000
Multivariate analysis				
AFP, ng/ml (≤20 versus >20)	1.101(0.826-1.469)	0.512	1.328(0.955-1.847)	0.091
GGT, U/L (≤54 versus >54)	1.628(1.239-2.141)	0.000	1.722(1.266-2.342)	0.001
Liver cirrhosis (no versus yes)	1.597(1.062-2.401)	0.024	NA	NA
Tumor size, cm (≤5 versus >5)	1.217(0.919-1.612)	0.171	1.476(1.086-2.007)	0.013
Tumor number (single versus multiple)	1.756(0.993-3.108)	0.053	1.347(0.703-2.580)	0.369
Microvascular invasion (no versus yes)	1.943(0.952-3.967)	0.068	1.030 (0.480-2.212)	0.939
Tumor differentiation (I + II versus III + IV)	1.405(1.038-1.901)	0.028	1.671(1.208-2.311)	0.002
TNM stage (I versus II III)	0.722(0.335-1.557)	0.406	1.375(0.601-3.147)	0.451
Sema3A (low versus high)	1.577(1.197-2.078)	0.001	1.912(1.401-2.610)	0.000
Intratumor CD68 (low versus high)	1.698(1.282-2.249)	0.000	1.968(1.441-2.689)	0.000
Intratumor CD163 (low versus high)	1.687(1.277-2.229)	0.000	1.876(1.375-2.561)	0.000
Combination of Sema3A and CD163*		0.000		0.000
I versus II	1.506(1.032-2.197)	0.034	1.606(1.037-2.486)	0.034
II versus III	1.386(0.987-1.947)	0.059	1.432(0.986-2.081)	0.060
I versus III	1.964(1.430-2.698)	0.000	2.296(1.593-3.309)	0.000

Cox proportional hazards regression model.

* I, Sema3A^low^/CD163^low^; II, Sema3A^low^/CD163^high^ and Sema3A^high^/CD163^low^; III, Sema3A^high^/CD163^high^. AFP, alpha-fetoprotein; GGT, gamma glutamyl transferase; TNM, tumor-node-metastasis.

HR, hazard ratio; CI, confidential interval; NA, not adopted.

## DISCUSSION

We found that compared with normal tissues, Sema3A was distinctly increased in carcinoma issues. Sema3A enhanced HCC cell ability to invade and metastasize in in vitro and in vivo conditions. Sema3A participates in both tumor progression [17, 26] and tumor suppression [14, 16]. However, no results have been previously published that describe the relationship between Sema3A and tumor metastatic potential. Our study revealed the presence of a positive correlation between Sema3A and the metastatic potential of HCC. Our results also indicated that Sema3A expression was clearly increased in tissues taken from patients who experienced tumor recurrence. Taken together, these results contribute to the understanding of the role of Sema3A in HCC metastasis and recurrence.

Sema3A is a chemoattractant for apical dendrites [4] and a chemorepellent for forcortical axons [27]. Sema3A presence is essential for the guidance of macrophages migrating into hypoxic niches [25]. However, its role in tumor progression and its relationship with TAMs in patients with HCC is largely unknown. An exciting correlation between Sema3A expression and TAMs was revealed by our study Sema3A acted as a chemoattractant for macrophages in vitro. Sema3A also had an essential role in TAM recruitment in xenograft tumor models. Finally, we used clinical HCC samples from independent cohorts of patients to validate the relationship between Sema3A and TAM infiltration (paraffin embedded tissues, Cohort 3, n=368). We found that the association between Sema3A expression and TAM number was statistically significant.

After migrating from the circulation and extravasating through the endothelium, monocytes differentiate into macrophages that aid in the resistance of pathogen invasion [28]. TAMs, in particular, have these characteristics. In many types of human carcinomas (e.g., HCC), the presence of extensive TAM infiltration correlates with poor patient prognosis [29–31]. These TAMs are well-known mediators of tumor invasion and metastasis. The results of our study indicated that compared with PLC/PRF/5-Mock-derived xenografts with low levels of Sema3A, larger tumors, more pulmonary metastases, and a greater number of TAMs could be detected in the HCCLM3-Mock-derived xenografts with high Sema3A levels. The inhibition of expression of Sema3A in the HCC cells reduced tumor volumes, pulmonary metastases, and TAM numbers. Sema3A overexpression resulted in adverse effects. These results indicated that tumor-derived Sema3A and the concomitant TAM infiltration promoted HCC progression in vivo.

To understand the role of Sema3A for the prediction of HCC patient outcome after curative resection, we used qRT-PCR assay to detect the expression of Sema3A mRNA in a cohort of 96 patients. The results indicated that patients with higher Sema3A levels had greater tumor recurrence rates. We also used immunohistochemistry assay to examine Sema3A protein levels in TMAs in cohort 3 (368 HCC patients). The multivariate analysis results indicated that Sema3A was a significant independent prognosticator for OS and TTR. Because the xenograft model results indicated that Sema3A was a chemoattractant for macrophages and a significant relationship between Sema3A expression and the number of TAMs was revealed in the clinical HCC samples, we used immunohistochemistry to survey the prognostic significance of TAMs using TMA. The results indicated that TAM number is a poor prognostic indicator for tumor recurrence after resection. Thus, the prognostic value of Sema3A expression and TAM number was determined for patients with this tumor. Patients were divided into three subgroups based on Sema3A expression and TAM number (Sema3A^low^/TAMs^low^, Sema3A^low^/TAMs^high^ or Sema3A^high^/TAMs^low^, and Sema3A^high^/TAMs^high^). A between-group comparison of prognosis revealed that patients with high Sema3A expression and high levels of TAM infiltration had a greater risk of recurrence after curative resection. The patients who had low Sema3A levels and low levels of TAM had the best prognosis. Sema3A expression had been viewed as an independent predictor for OS and TTR, but the predictive value of Sema3A^high^/TAMs^high^ was greater than that of Sema3A^high^ or TAMs^high^ alone.

Dynamic interactions between tumor cells and between these cells and elements of the tumor inflammatory environment (e.g., inflammatory cells and inflammatory mediators) lead to tumor growth and dissemination [32]. Our results indicated that Sema3A is a key mediator of macrophage recruitment into the tumor microenvironment. However, given the complexity of the tumor microenvironment, this environment may also mediate the effects of Sema3A on tumor progression. The localization of hypoxia-induced TAMs into hypoxic tumor areas is controlled by Sema3A [25]. We will, therefore, further investigate the effects of the tumor microenvironment on Sema3A.

Taken together, our results indicated that Sema3A enhanced cell proliferation, invasion, and TAM infiltration in HCC patients. Sema3A, alone or in combination with TAMs, merits more study as a novel prognostic marker for patients who undergo tumor resection with curative intent. These results also suggested that Sema3A is a potential therapeutic target.

## MATERIALS AND METHODS

### Cell lines and animals

Our institute established HCC cell lines (MHCC97L, MHCC97H, and HCCLM3) that have identical genetic backgrounds and between-line stepwise metastatic potential [20]. These cell lines and low-metastatic human HCC cell lines (HepG2, PLC/PRF/5, and Huh7; Institute of Biochemistry and Cell Biology, Chinese Academy of Sciences, Shanghai, China) were all preserved and validated.

Male, nu/nu mice (BALB/c, 4 to 6 weeks of age, Shanghai Institute of Material Medicine, Chinese Academy of Science) were maintained in specific pathogen-free conditions and were provided with adequate food and water. All animal care procedures followed the criteria outlined in the “Guide for the Care and Use of Laboratory Animals” (National Institutes of Health, Washington, DC, USA).

### Patients and follow-up

Three independent cohorts (total of 527 HCC patients) were enrolled in the study. Cohort 1 consisted of 96 patients (45 patients with, and 51 patients without, recurrence). From the patients in Cohort 1, snap-frozen tissue samples were collected consecutively when they underwent curative resection at our institute during the 1 August to 31 December 2008 period. The second cohort (Cohort 2) consisted of 63 patients. The snap-frozen tumor and paired neighboring cirrhotic liver (i.e., non-tumor), were consecutively collected when they underwent resection with curative intent at our institute during the 1 January to 30 June 2007 period. Samples from the third cohort (Cohort 3, n=368, tissues embedded in paraffin) were randomly collected when patients underwent resection with curative intent in 2006. All patients were postsurgically monitored until 15 March 2013. The histopathological diagnosis was made using World Health Organization criteria. The Edmondson and Steiner classification was used to determine the histological tumor differentiation grade [33]. Assessment of tumor stage was based on the 2010 International Union Against Cancer tumor-node-metastasis classification system. To estimate liver function, the Child-Pugh scoring system was used. Written informed consent was obtained from each patient. The Zhongshan Hospital Research Ethics Committee approved the study. Patient follow-up after surgery was performed as previously described [24, 34]. Overall survival (OS) time was the interval between the surgery date and the date of death, or the date of the last patient observation. Time to recurrence (TTR) [35] was the defined as the interval of time between surgery and the diagnosis of any relapse, including intrahepatic recurrence and extrahepatic metastasis.

### RNA isolation and qRT-PCR

Total RNA was isolated from cell lines and frozen tumor specimens collected from all three cohorts described above (Trizol reagent; Invitrogen, Carlsbad, CA, USA) per the manufacturer's instructions. mRNA expression of Semaphorin 3A (Sema3A) in the cell lines, 159 carcinoma tissue samples, and 63 samples of adjacent normal liver tissues was measured using qRT-PCR (ABI7900HT Fast Real-Time PCR System, Applied Biosystems, Foster City, CA, USA). qRT-PCR was determined using a SYBR PrimeScript RT-PCR Kit (Takara Bio, Shiga, Japan), per the manufacturer's instructions. For an internal control, the GAPDH primers were: Sema3A (Genbank NM_006080) forward primer 5′-TTCATTAGTGCCCACCTCATCTCA-3′ and reverse primer 5′-TTCTGTGCCCTCCAAAGTCATTC-3′ and GAPDH (Genbank NM_002046) forward primer 5′-GGTATGACAACGAATTTGGC-3′ and reverse primer 5′-GAGCACAGGGTACTTTATTG -3′. Relative mRNA levels were calculated using the equation: 2^−ΔCt^ [ΔCt = Ct (Sema3A) - Ct (GAPDH)]. qRT-PCR was used to determine Sema3A expression in cohort 1 patients (n=96) and cohort 2 patients (n=63). In subsequent analyses, the cut-off value was the median Sema3A mRNA level for the group. Patients with Sema3A mRNA levels greater and less than the cut-off value were defined as Sema3A^high^ and Sema3A^low^, respectively. All evaluations were performed in triplicate.

### Western blot analysis

Western blot analysis was used to detect Sema3A protein expression in the cell lines. Briefly, total protein was extracted in lysis buffer (45 min, on ice). Denatured proteins were resolved by SDS-PAGE, then transferred to polyvinylidene difluoride membranes. The membranes were washed, blocked, and incubated with specific primary antihuman antibodies against Sema3A (1:250, Abnova, Taipei, Taiwan), and GAPDH (1:5000; Millipore, Bedford, MA, USA). The appropriate horseradish peroxidase-conjugated secondary antibodies were added, and enhanced chemiluminescence assay was used to visualize the bands.

### Enzyme-Linked immunosorbent assay (ELISA)

The cell culture supernatant Sema3A level was determined using a Semaphorin 3A ELISA Kit (LifeSpan BioSciences, Seattle, WA, USA), according to the manufacturer's instructions. The absorbance (λ=450 nm) was measured using a Microplate Absorbance Spectrophotometer (Bio-Rad, Hercules, CA, USA). An absorbance versus Sema3A concentration (in the standard wells) curve was then plotted.

### Cell transfection and clone selection

Ubi-MCS-Luc-IRES-Puromycin-Sema3A and mU6-MCS-Ubi-luc-shRNA-Sema3A lentiviral vectors were acquired from Shanghai GeneChem Co. (Shanghai, China). Ubi-MCS-Luc-IRES-Puromycin-Sema3A was transfected into PLC/PRF/5 and Ubi-MCS-Luc-IRES-Puromycin lentiviral vectors were used as controls. mU6-MCS-Ubi-Luc-shRNA-Sema3A was transfected into HCCLM3 and mU6-MCS-Ubi-Luc was used as a control. Stably transfected clones were validated for Sema3A using qRT-PCR.

### Cell proliferation assay, cell migration, and matrigel invasion assays

Cells were seeded into a 96-well plate (2000 cells/well). At various points in time (24, 48, 72, and 96 hours), 10 μL CCK-8 solution (Dojindo, Tokyo, Japan) were added to each well, and each plate was incubated for an additional 2 hours. The absorbance value (λ=450 nm) was used to calculate the number of viable cells in each well. Each experiment was performed in triplicate.

The scratch wound assay was used to evaluate cell migration. Cells were cultured (2 days) to form a tight monolayer and then were serum-starved for 16 hours. A 10-μL plastic pipette tip was used to wound the cell monolayer. To remove cell debris, the remaining cells were washed (twice) and incubated at 37°C with normal serum-containing culture medium. Photographs of cells that migrated to the wound front were taken at 24, 48, and 72 hours using an inverted microscope (Leica, Hesse, Germany). Migration capacity was quantified using measurements of the percent of open area.

Transwells (24-well, 8-μm pore size; Millipore, Billerica, MA, USA) precoated with Matrigel (BD Biosciences, Franklin Lakes, NJ, USA) were used for the cell invasion assays. A total of 1×10^5^ cells were seeded into 100 μL Dulbecco's modified Eagle medium (DMEM) with 1% fetal bovine serum. The cells were then placed in the upper chamber and 600 μL DMEM with 10% fetal bovine serum was added to the lower chamber. The upper-chamber cells were removed after a 48-hour incubation period and those on the lower surface of the membrane were fixed using paraformaldehyde (4%). The cells were then stained (Giemsa stain), counted (200× magnification), and photographed.

### In vivo assays for tumor growth and metastasis

The inoculated cells were resuspended in a 1:1 (v/v) solution of DMEM. Each nude mouse then received a subcutaneous injection of the solution into the upper left flank area. The subcutaneous tumor that developed was removed when it measured approximately 1 cm diameter. After removal, each tumor was cut into small pieces (approximately 2×2×2 mm^3^ volume), and then transplanted into the liver of a nude mouse. Each mouse was examined once every 5 days and was euthanized at 5 weeks after the transplant. Tumor volume (mm^3^) was calculated as: V = ab2/2; a and b were the largest and smallest tumor diameters measured at necropsy, respectively [22]. The lungs of each mouse were removed, embedded in paraffin blocks, and serially sectioned (5 μm) for histopathologic analysis. Metastasis was classified into one of four grades based on the tumor cell number present at the maximal section of each metastatic lesion (grade I, ≤20; grade II, 20–50; grade III, 50–100; and grade IV, >100). After an intraperitoneal injection of 150 mg/kg D-luciferin (Promega, Madison, WI, USA), each isoflurane-anesthetized animal was imaged using the IVIS system (IVIS, Taranto, Italy). Living Image software (Caliper LifeSciences, Waltham, MA, USA) was used to quantify the results.

### Human macrophage isolation and preparation

To isolate macrophages, the human liver tissue was minced and digested using 0.05% collagenase IV, 0.002% DNase I (Sigma-Aldrich, St. Louis, MO, USA), and 20% fetal bovine serum (FBS) (Gibco, Waltham, MA, USA) at 37°C for 1 hour. The dissociated cells were passed through a 150-μm mesh filter and separated using Percoll centrifugation. The EasySep™ Human CD14 Positive Selection Kit (Stemcell Technologies, Vancouver, Canada) was used to isolate CD14+ cells from the mononuclear cell suspension.

### Macrophage chemotaxis assay

A Transwell system (Corning, Corning, NY, USA) and 5-μm polycarbonate membranes was used for the macrophage chemotaxis assay. Briefly, recombinant human Sema3A at various concentration, or conditioned medium (CM) separated from HCC cells, with or without control Ab (1μg/mL) or anti-Sema3A antibody (1μg/mL) were added to the bottom wells. Macrophages suspended in RPMI 1640 in the presence of 2% FBS (2×10^5^ cells/100 μL) were added to the top wells and incubated for 24 h at 37°C, 5% CO_2_. After incubation, the upper-chamber macrophages were removed, fixed, stained, and photographed. The chemotactic index (the ratio of the number of macrophages that migrated to different amboceptor-containing wells divided by the number of macrophages that migrated to RPMI 1640 alone) was then calculated.

### Tissue microarray and immunohistochemistry

Tissue microarray construction was conducted as previously described [36]. Two 1 mm diameter core biopsies were removed from the donor blocks. They were then transferred to defined array positions in the recipient paraffin block. Three tissue microarray (TMA) blocks (368 cases, Cohort 3) were constructed. We used the avidin-biotin-peroxidase complex method to perform the immunohistochemical staining. Briefly, rehydration and microwave antigen retrieval were performed first. Antibodies against human Sema3A (1:250, PAB7888; Abnova), F4/80 (1:250, ab100790; Abcam), CD163 (1:200, ab126756; Abcam) or CD68 (1:200, ab955; Abcam) were then applied to the slides, and incubated at 4°C overnight. The secondary antibody incubation (GK500705, Gene Tech, China) was then performed at 37°C for 30 min using 3,3′-diaminobenzidine and a Mayer's hematoxylin counter-stain.

### Immunohistochemical variable evaluation

Results from immunohistochemical staining were viewed independently by three investigators. Each investigator was blinded to the patients’ demographics and medical history. Three representative fields were photographed using Leica QWin Plus v3 software under high-power magnification (200×). Image-Pro Plus v6.2 software (Media Cybernetics, Inc., Bethesda, MD, USA) was used to count the Sema3A density. The cut-off value used for subsequent analyses was the median value for Sema3A density. Patients with a Sema3A density value greater than or less than the median value were defined as Sema3A^high^ or Sema3A^low^, respectively. For the CD68 staining result interpretation in the TMA, we counted positive cells in each 1-mm-diameter cylinder as previously described [20]. Unless indicated otherwise, in subsequent analyses the cut-off values were the median values.

## References

[1] Ikai I, Arii S, Kojiro M, Ichida T, Makuuchi M, Matsuyama Y, Nakanuma Y, Okita K, Omata M, Takayasu K, Yamaoka Y (2004). Reevaluation of prognostic factors for survival after liver resection in patients with hepatocellular carcinoma in a Japanese nationwide survey. Cancer.

[2] Tang ZY, Ye SL, Liu YK, Qin LX, Sun HC, Ye QH, Wang L, Zhou J, Qiu SJ, Li Y, Ji XN, Liu H, Xia JL, Wu ZQ, Fan J, Ma ZC (2004). A decade's studies on metastasis of hepatocellular carcinoma. Journal of cancer research and clinical oncology.

[3] Torre LA, Bray F, Siegel RL, Ferlay J, Lortet-Tieulent J, Jemal A (2015). Global cancer statistics, 2012. CA.

[4] Polleux F, Morrow T, Ghosh A (2000). Semaphorin 3A is a chemoattractant for cortical apical dendrites. Nature.

[5] Chen G, Sima J, Jin M, Wang KY, Xue XJ, Zheng W, Ding YQ, Yuan XB (2008). Semaphorin-3A guides radial migration of cortical neurons during development. Nature neuroscience.

[6] Casazza A, Finisguerra V, Capparuccia L, Camperi A, Swiercz JM, Rizzolio S, Rolny C, Christensen C, Bertotti A, Sarotto I, Risio M, Trusolino L, Weitz J, Schneider M, Mazzone M, Comoglio PM (2010). Sema3E-Plexin D1 signaling drives human cancer cell invasiveness and metastatic spreading in mice. The Journal of clinical investigation.

[7] Ito M, Ito G, Kondo M, Uchiyama M, Fukui T, Mori S, Yoshioka H, Ueda Y, Shimokata K, Sekido Y (2005). Frequent inactivation of RASSF1A, BLU, and SEMA3B on 3p21. 3 by promoter hypermethylation and allele loss in non-small cell lung cancer. Cancer letters.

[8] Karayan-Tapon L, Wager M, Guilhot J, Levillain P, Marquant C, Clarhaut J, Potiron V, Roche J (2008). Semaphorin, neuropilin and VEGF expression in glial tumours: SEMA3G, a prognostic marker?. British journal of cancer.

[9] Miyato H, Tsuno NH, Kitayama J (2012). Semaphorin 3C is involved in the progression of gastric cancer. Cancer science.

[10] Martin-Satue M, Blanco J (1999). Identification of semaphorin E gene expression in metastatic human lung adenocarcinoma cells by mRNA differential display. Journal of surgical oncology.

[11] Tseng CH, Murray KD, Jou MF, Hsu SM, Cheng HJ, Huang PH (2011). Sema3E/plexin-D1 mediated epithelial-to-mesenchymal transition in ovarian endometrioid cancer. PloS one.

[12] Drenberg CD, Livingston S, Chen R, Kruk PA, Nicosia SV (2009). Expression of Semaphorin 3F and Its Receptors in Epithelial Ovarian Cancer, Fallopian Tubes, and Secondary Mullerian Tissues. Obstetrics and gynecology international.

[13] Tischoff I, Markwarth A, Witzigmann H, Uhlmann D, Hauss J, Mirmohammadsadegh A, Wittekind C, Hengge UR, Tannapfel A (2005). Allele loss and epigenetic inactivation of 3p21. 3 in malignant liver tumors. International journal of cancer Journal international du cancer.

[14] Staton CA, Shaw LA, Valluru M, Hoh L, Koay I, Cross SS, Reed MW, Brown NJ (2011). Expression of class 3 semaphorins and their receptors in human breast neoplasia. Histopathology.

[15] Barresi V, Vitarelli E, Cerasoli S (2009). Semaphorin3A immunohistochemical expression in human meningiomas: correlation with the microvessel density. Virchows Archiv : an international journal of pathology.

[16] Yoshikawa Y, Sato A, Tsujimura T, Morinaga T, Fukuoka K, Yamada S, Murakami A, Kondo N, Matsumoto S, Okumura Y, Tanaka F, Hasegawa S, Hashimoto-Tamaoki T, Nakano T (2011). Frequent deletion of 3p21. 1 region carrying semaphorin 3G and aberrant expression of the genes participating in semaphorin signaling in the epithelioid type of malignant mesothelioma cells. International journal of oncology.

[17] Muller MW, Giese NA, Swiercz JM, Ceyhan GO, Esposito I, Hinz U, Buchler P, Giese T, Buchler MW, Offermanns S, Friess H (2007). Association of axon guidance factor semaphorin 3A with poor outcome in pancreatic cancer. International journal of cancer Journal international du cancer.

[18] Bishop WR, Bell RM (1988). Functions of diacylglycerol in glycerolipid metabolism, signal transduction and cellular transformation. Oncogene research.

[19] Nagaoka K, Fujii K, Zhang H, Usuda K, Watanabe G, Ivshina M, Richter JD (2015). CPEB1 mediates epithelial-to-mesenchyme transition and breast cancer metastasis. Oncogene.

[20] Zhou SL, Dai Z, Zhou ZJ, Wang XY, Yang GH, Wang Z, Huang XW, Fan J, Zhou J (2012). Overexpression of CXCL5 mediates neutrophil infiltration and indicates poor prognosis for hepatocellular carcinoma. Hepatology.

[21] Galluzzi L, Vitale I, Senovilla L, Olaussen KA, Pinna G, Eisenberg T, Goubar A, Martins I, Michels J, Kratassiouk G, Carmona-Gutierrez D, Scoazec M, Vacchelli E, Schlemmer F, Kepp O, Shen S (2012). Prognostic impact of vitamin B6 metabolism in lung cancer. Cell reports.

[22] Zhou SL, Dai Z, Zhou ZJ, Chen Q, Wang Z, Xiao YS, Hu ZQ, Huang XY, Yang GH, Shi YH, Qiu SJ, Fan J, Zhou J (2014). CXCL5 contributes to tumor metastasis and recurrence of intrahepatic cholangiocarcinoma by recruiting infiltrative intratumoral neutrophils. Carcinogenesis.

[23] Zhou SL, Zhou ZJ, Hu ZQ, Li X, Huang XW, Wang Z, Fan J, Dai Z, Zhou J (2015). CXCR2/CXCL5 axis contributes to epithelial-mesenchymal transition of HCC cells through activating PI3K/Akt/GSK-3beta/Snail signaling. Cancer letters.

[24] Zhou ZJ, Dai Z, Zhou SL, Hu ZQ, Chen Q, Zhao YM, Shi YH, Gao Q, Wu WZ, Qiu SJ, Zhou J, Fan J (2014). HNRNPAB induces epithelial-mesenchymal transition and promotes metastasis of hepatocellular carcinoma by transcriptionally activating SNAIL. Cancer research.

[25] Casazza A, Laoui D, Wenes M, Rizzolio S, Bassani N, Mambretti M, Deschoemaeker S, Van Ginderachter JA, Tamagnone L, Mazzone M (2013). Impeding macrophage entry into hypoxic tumor areas by Sema3A/Nrp1 signaling blockade inhibits angiogenesis and restores antitumor immunity. Cancer cell.

[26] Biankin AV, Waddell N, Kassahn KS, Gingras MC, Muthuswamy LB, Johns AL, Miller DK, Wilson PJ, Patch AM, Wu J, Chang DK, Cowley MJ, Gardiner BB, Song S, Harliwong I, Idrisoglu S (2012). Pancreatic cancer genomes reveal aberrations in axon guidance pathway genes. Nature.

[27] Polleux F, Giger RJ, Ginty DD, Kolodkin AL, Ghosh A (1998). Patterning of cortical efferent projections by semaphorin-neuropilin interactions. Science.

[28] Murray PJ, Wynn TA (2011). Protective and pathogenic functions of macrophage subsets. Nature reviews Immunology.

[29] De Palma M, Lewis CE (2013). Macrophage regulation of tumor responses to anticancer therapies. Cancer cell.

[30] Yeung OW, Lo CM, Ling CC, Qi X, Geng W, Li CX, Ng KT, Forbes SJ, Guan XY, Poon RT, Fan ST, Man K (2015). Alternatively activated (M2) macrophages promote tumour growth and invasiveness in hepatocellular carcinoma. Journal of hepatology.

[31] Yan W, Liu X, Ma H, Zhang H, Song X, Gao L, Liang X, Ma C (2015). Tim-3 fosters HCC development by enhancing TGF-beta-mediated alternative activation of macrophages. Gut.

[32] Lazennec G, Richmond A (2010). Chemokines and chemokine receptors: new insights into cancer-related inflammation. Trends in molecular medicine.

[33] Wittekind C (2006). [Pitfalls in the classification of liver tumors]. Pathologe.

[34] Zhou ZJ, Dai Z, Zhou SL, Fu XT, Zhao YM, Shi YH, Zhou J, Fan J (2013). Overexpression of HnRNP A1 promotes tumor invasion through regulating CD44v6 and indicates poor prognosis for hepatocellular carcinoma. International journal of cancer Journal international du cancer.

[35] Llovet JM, Di Bisceglie AM, Bruix J, Kramer BS, Lencioni R, Zhu AX, Sherman M, Schwartz M, Lotze M, Talwalkar J, Gores GJ (2008). Design and endpoints of clinical trials in hepatocellular carcinoma. J Natl Cancer Inst.

[36] Dai Z, Zhou SL, Zhou ZJ, Bai DS, Xu XY, Fu XT, Chen Q, Zhao YM, Zhu K, Yu L, Yang GH, Wang Z, Wu WZ, Zhou J, Fan J (2014). Capn4 contributes to tumour growth and metastasis of hepatocellular carcinoma by activation of the FAK-Src signalling pathways. The Journal of pathology.

